# Non-cancerous CT findings as predictors of survival outcome in advanced non-small cell lung cancer patients treated with first-generation EGFR-TKIs

**DOI:** 10.1371/journal.pone.0313577

**Published:** 2025-02-05

**Authors:** Pakorn Prakaikietikul, Pattraporn Tajarenmuang, Phumiphat Losuriya, Natee Ina, Thanika Ketpueak, Thanat Kanthawang

**Affiliations:** 1 Department of Radiology, Faculty of Medicine, Chiang Mai University, Chiang Mai, Thailand; 2 Division of Pulmonary, Critical Care, and Allergy, Department of Internal Medicine, Faculty of Medicine, Chiang Mai University, Chiang Mai, Thailand; 3 Radiological Technology Division, Department of Radiology, Faculty of Medicine, Prince of Songkla University, Songkla, Thailand; 4 Division of Oncology, Department of Internal Medicine, Faculty of Medicine, Chiang Mai University, Chiang Mai, Thailand; Maria Sklodowska-Curie National Research Institute of Oncology Krakow, POLAND

## Abstract

**Purpose:**

To identify non-cancerous factors from baseline CT chest affecting survival in advanced non-small cell lung cancer (NSCLC) treated with first-generation Epidermal Growth Factor Receptor-Tyrosine Kinase Inhibitors (EGFR-TKIs).

**Methods:**

Retrospective study of 172 advanced NSCLC patients treated with first-generation EGFR-TKIs as a first-line systemic treatment (January 2012 to September 2022). Baseline CT chest assessed visceral/subcutaneous fat (L1 level), sarcopenia, and myosteatosis (multiple levels), main pulmonary artery (MPA) size, MPA to aorta ratio, emphysema, and bone mineral density. Cox regression analyzed prognostic factors at 18-month outcome.

**Results:**

Median overall survival was 17.57 months (14.87–20.10) with 76 (44.19%) patients died at 18 months. Deceased had lower baseline BMI (21.10 ± 3.44) vs. survived (23.25 ± 4.45) (p < 0.001). Univariable analysis showed 5 significant prognostic factors: low total adiposity with/without cutoff [HR 2.65 (1.68–4.18), p < 0.001; 1.00 (0.99–1.00), p = 0.006;], low subcutaneous adipose tissue (SAT) with/without cutoff [HR 1.95 (1.23–3.11), p = 0.005; 0.99 (0.98–0.99), p = 0.005], low SAT index (SATI) with/without cutoff [1.74 (1.10–2.78), p = 0.019; 0.98 (0.97–0.99), p = 0.003], high VSR [1.67 (1.06–2.62), p = 0.026], and high MPA size with/without cutoff [2.23 (1.23–4.04), p = 0.005; 1.09 (1.04–1.16), p = 0.001]. MPA size, MPA size > 29 mm, and total adiposity ≤85 cm^2^ remained significant in multivariable analysis, adjusted by BMI [HR 1.14 (1.07–1.21), p < 0.001; 3.10 (1.81–5.28), p < 0.001; 3.91 (1.63–9.40), p = 0.002]. There was no significant difference of sarcopenic and myosteatotic parameters between the two groups.

**Conclusion:**

In advanced EGFR-mutated NSCLC patients, assessing pre-treatment prognosis is warranted to predict the survival outcome and guide decision regarding EGFR-TKI therapy. Enlarged MPA size, low total adiposity, and low subcutaneous fat (lower SAT, lower SATI, and higher VSR) are indicators of poor survival. Large MPA size (>29 mm) or low total adiposity (≤85 cm^2^) alone predict 18-month death.

## Introduction

Lung cancer is the leading cause of death globally, accounting for about 1.79 million deaths, or 18% of all cancer-related deaths [1]. Non-small cell lung cancer (NSCLC) makes up 80–90% of lung cancers, with adenocarcinoma being the most common subtype [2]. Recently, treatment for NSCLC has shifted from conventional chemotherapy to targeted therapies, driven by molecular alterations. Epidermal growth factor receptor (EGFR) mutations, found in 32.3% to 50.7% of NSCLC patients in Asia [3–5], are commonly treated with EGFR tyrosine kinase inhibitors (TKIs). A meta-analysis showed that EGFR-TKIs significantly improve overall and progression-free survival in patients with EGFR-mutated NSCLC [6].

Prognosis in NSCLC is influenced by both cancer-related and host factors [7]. Chest computed tomography (CT) scans, routinely performed before treatment, have the potential to reveal both types of prognostic factors. While many studies have focused solely on cancer-related factors, non-cancerous host factors are also important to reflect overall conditions of the patients and treatment tolerance [7]. In a few studies, main pulmonary artery (MPA) size, underlying lung diseases and body composition-such as muscle mass and body fat adiposity-have been shown to affect patient outcome and treatment toxicity [7–10]. However, these previous studies have predominantly included lung cancer patients receiving various treatments, without a specific focus on EGFR-TKI treatment [11]. Studies on body composition in lung cancer patients are sparse and often focus on sarcopenia through muscle volume measurement, while neglecting infiltration of fat in the muscle or also called myosteatosis [11]. Sarcopenia is usually assessed by measuring muscle volume at the L3 vertebral level, which is not typically included in chest CT scans, limiting its practical use [12–14]. However, measuring muscle volume at the thoracic and upper lumbar levels has shown promise in predicting prognosis in several cancers [12,13].

At the diagnosis, non-cancerous factors including body mass index (BMI), sarcopenia, adiposity, and myosteatosis have the potential to predict patients who may have unfavorable outcomes to the first-generation EGFR-TKIs and may benefit from initiating the higher-generation TKIs or combination treatment with chemotherapy or anti-angiogenesis agents [15–17]. The measurement of muscle volume at the thoracic and upper lumbar spine levels is desirable, as it routinely included in chest CT scans without adding cost or patient’s radiation exposure. Additionally, anthropometric measurements of body composition, including adiposity, myosteatosis and sarcopenic assessment, provide insights into patients’ status and can guide early preventive strategies, such as improving nutritional status [11].

This study aims to identify baseline non-cancerous CT factors associated with survival outcomes in advanced NSCLC patients receiving first-generation EGFR-TKIs. The secondary objective is to determine the proper level for evaluating sarcopenia and myosteatosis in chest CT scans to predict survival outcomes.

## Materials and methods

### Patients and study design

**Fig 1:**
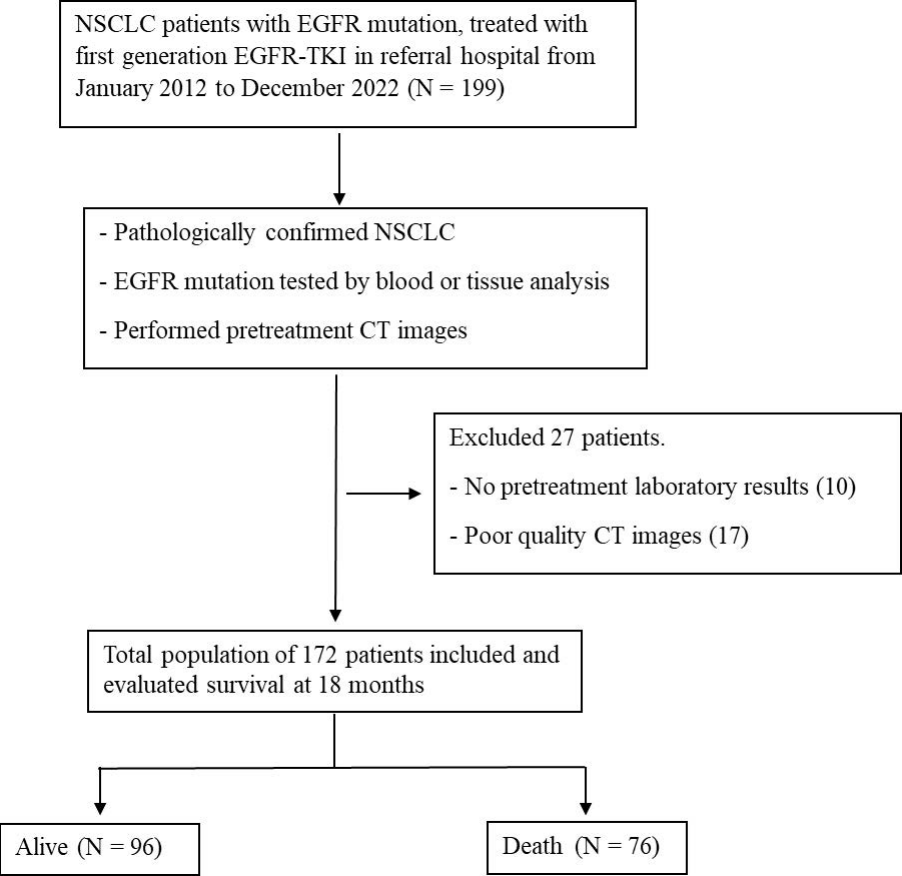
The study flow and patient selection. NSCLC; non-small cell lung cancer, EGFR; epidermal growth factor receptor, TKI; tyrosine kinase inhibitor, CT; computed tomography.

A retrospective study reviewed contrast-enhanced chest CT scans of patients diagnosed with advanced NSCLC with EGFR mutation who received first-generation EGFR-TKIs treatment as the first-line systemic treatment at Chiang Mai University Hospital from January 2012 to September 2022, yielding 199 cases. We assessed the database for data collection for this study from 01/01/2024 to 13/03/2024. Exclusion criteria included the absence of available medical records (10 patients) and pretreatment CT images (17 patients), leaving 172 cases included in this study (Fig 1). The institutional review board approved this study (MED-2566-09423) and waived the requirement for informed consent due to its retrospective nature.

The pretreatment data were collected from the electronic medical records, including age, sex, body weight, height, BMI, The Eastern Cooperative Oncology Group (ECOG) performance status, smoking status, underlying disease, presence of metastasis, number of metastasis, staging and pathologic subtypes (Supplementary Material S1 File). The primary endpoint of this study is an overall survival (OS) rate at 18 month as mentioned in prior literature of Thai population [17] (Supplementary Material S1 File).

### CT analysis

All patients underwent post-contrast CT scanning of the chest and upper abdomen using one of two machines: the SOMATOM® Definition (Siemens Healthcare, Forchheim, Germany) or the SOMATOM® Force (Siemens Healthcare, Forchheim, Germany). Scans were performed with 120 kVp and modified mAs for reduced radiation. A collimation of 128 × 0.6 mm with the z-flying focal spot technique was used. Image series were acquired in the craniocaudal direction, with patients in a supine position and both arms extended above the head. The anatomic range extended from the thoracic inlet to the L2 vertebra. Axial images from the post-contrast CT at each level were used for evaluation.

Non-cancerous CT findings included amount of sarcopenia and myosteatosis, visceral and subcutaneous fat, MPA size and MPA to aorta ratio, degree of emphysema, bone mineral density (BMD). Apart from sarcopenic and myosteatotic measurement, the remaining factors were reviewed and measured by two radiologists with 3 and 9 years of experience (P.P. and T.K., respectively). In cases of disagreement, the final decision was obtained by consensus for categorical data and used mean valued for continuous data.

The CT images for sarcopenia and myosteatosis were measured at multiple anatomical landmarks, including the first lumbar vertebra (L1), the twelfth thoracic vertebra (T12), T10, T5, and the aortic arch, as referenced in previous literature [18,19]. The CT images at each level were carefully chosen and archived as Digital Imaging and Communications in Medicine (DICOM) data. All DICOM data calculated body composition using in-house software developed by MATLAB (The MathWorks, Natick, MA, USA) and freeware Python 3.6.13 (Anaconda, Inc.), to generate the measurement model based on neural network architecture also known as UNet. The valid accuracy of the model was 99.17% and validity of the intersect over union co-efficiency was 89.40% [20].

For sarcopenia and myosteatosis parameters, we used L1 as the reference standard for statistical analysis, as recommended by previous literature [19], and compared the results with those from other levels. All muscles were measured at the T10, T12, and L1 levels, while only the chest wall muscles (excluding the paraspinal muscles) were measured at the T5 and aortic arch levels. The analysis included three parameters. Firstly, muscular surface area (MSA) defined as a surface area of the muscles at evaluated levels in centimeter^2^ (cm^2^). Secondly, skeleton muscle index (SMI) was MSA (cm^2^) divided by the square of patient’s height (meter^2^ or m^2^). Finally, the average Hounsfield Units (HU) in the areas of the muscles of interest at each level were used to calculate the mean attenuation or skeletal muscle density (SMD) based on the pixels’ areas with attenuation between −29 and + 150 HU [21]. We decided to use specific cutoffs for each parameter at the L1 level, based on prior studies in the Asian population, due to the influence of ethnicity on body composition [19,22]. Sarcopenia at L1 level was defined as MSA < 116.2 cm^2^ for males and < 73.7 cm^2^ for females, and SMI < 39.2 cm^2^/m^2^ for males and < 27.5 cm^2^/m^2^ for females [19]. Myosteatosis at the L1 level was defined as SMD < 37.4 HU for males and <33.1 HU for females [23].

**Fig 2:**
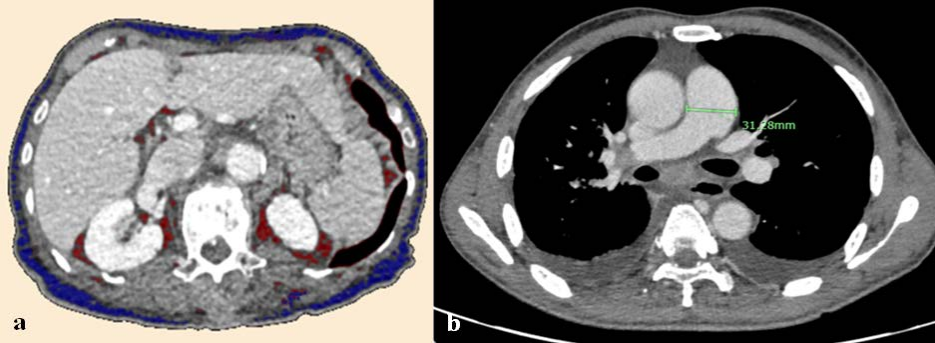
A 54-year-old man with a BMI of 18.03 kg/m^2^ who passed away after 3 months of EGFR-TKI treatment. The axial baseline post-contrast CT scan (a) at L1 level showed SAT = 0.53 cm^2^ (blue label) and VAT = 4.58 cm^2^ (red label) (b) at the level of the pulmonary artery bifurcation showed MPA size = 31.3 mm. BMI; body mass index, EGFR; epidermal growth factor receptor, TKI; tyrosine kinase inhibitor, CT; computed tomography, SAT; subcutaneous adipose tissue, cm; centimeters, VAT; visceral adipose tissue, MPA; main pulmonary artery, mm; millimeters.

**Fig 3:**
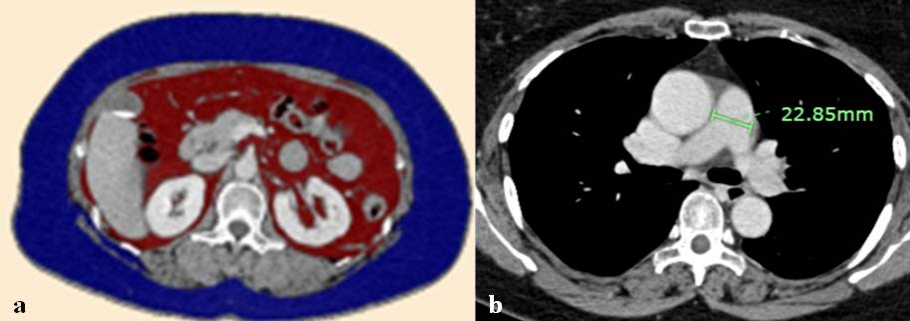
A 64-year-old woman with a BMI of 25.33 kg/m^2^ who survived after 18 months of EGFR-TKI treatment. The axial baseline post-contrast CT scan (a) at L1 level showed SAT = 275.24 cm^2^ (blue label) and VAT = 124.54 cm^2^ (red label) (b) at the level of the pulmonary artery bifurcation showed MPA size = 22.9 mm. BMI; body mass index, EGFR; epidermal growth factor receptor, TKI; tyrosine kinase inhibitor, CT; computed tomography, SAT; subcutaneous adipose tissue, cm; centimeters, VAT; visceral adipose tissue, MPA; main pulmonary artery, mm; millimeters.

For subcutaneous and visceral fat measurement, an axial plane through the L1 spine was measured in total fat areas, subcutaneous fat area, visceral fat area, and girth using a semiautomatic software program (Synapse Vincent 3D Version 4.4, Fujifilm Medical, Tokyo, Japan) (Figs 2 and 3). Abdominal girth represents abdominal circumference at interested level in centimeter (cm) [24]. The acceptable attenuation ranges of − 190 to − 30 HU for subcutaneous fat and − 150 to − 50 HU for visceral fat were used [25]. The representation of combined subcutaneous and visceral fat as total adiposity was derived from previous study [26]. Subcutaneous adipose tissue (SAT, cm^2^) and visceral adipose tissue (VAT, cm^2^) were normalized by height squared to calculate the subcutaneous adipose tissue index (SATI) and visceral adipose tissue index (VATI). Visceral-to-subcutaneous fat ratio (VSR) was also calculated by dividing VAT by SAT. Cutoff values of these measurements were also referenced from prior studies in Asian population, measured at L3 level, due to the effect of ethnicity on body composition [21,25,27]. Low SAT was defined as SAT ≤ 45 cm^2^, VAT ≤ 75 cm^2^, based on a prior study conducted in patients with NSCLC [28]. Low SATI was defined as SATI ≤ 40 cm^2^/m^2^ or ≤ 30 cm^2^/m^2^ in males and females. Low VATI was defined as VATI ≤ 44 cm^2^/m^2^ or ≤35 cm^2^/m^2^ in males and females [25]. High VSR was defined as VSR > 1.33 or > 0.93 in males and females [21]. The cutoff for low abdominal girth is ≤90 cm in men and ≤80 cm in women [29]. There has been no available cutoff for total adiposity.

The pulmonary artery size was measured of the MPA at the level of its bifurcation in an axial image (Figs 2 and 3). The size of the aorta was also measured at the same level. The MPA to aorta ratio was then calculated by dividing the size of the MPA by the size of the aorta [30]. The cutoff value for pulmonary hypertension is when the MPA size is more than 29 millimeters (mm) or the MPA to aorta ratio is greater than 1 [31].

For assessing the degree of COPD, we employed a simple visual assessment that can be easily performed, ranging from none to very severe emphysema [32]. “None” indicates no emphysema present. “Mild” signifies less than 25% of emphysema compared to the whole lung volume. “Moderate” indicates 25–50% of emphysema compared to the whole lung volume. “Severe” denotes 50–75% of emphysema compared to the whole lung volume. Finally, “very severe” indicates more than 75% of emphysema compared to the whole lung volume. There are three types of pulmonary emphysema [33]: centrilobular emphysema, which involves lung parenchyma at the center of the respiratory bronchiole; paraseptal emphysema, which involves the lung parenchyma adjacent to the pleura; and panlobular emphysema, which involves lung parenchyma throughout the pulmonary lobules.

Bone Mineral Density (BMD) was measured at T12 vertebral body, using ROI tool with a diameter of 10–15 mm at mid vertebral body. In cases of bony lesions at the T12 level, measurements were taken at the vertebral body one level above or below instead. A Hounsfield Unit value of 90 is used as the cutoff point for low BMD, indicating osteopenia [34].

### Data analysis

Statistical analysis was performed using STATA Statistical software version 16. The critical level of statistical significance set at p-value less than 0.05. Data will be summarized descriptively using mean, SD (for normally distributed data) and median, interquartile range (IQR) (for non- normally distributed data) for continuous data, counts and percentages for categorical data. Group differences were calculated with T-test or Wilcoxon ranksum test for continuous data, and Fisher exact test for categorical data. Due to the lack of a gold standard for cutoffs and the impact of ethnicity on anthropometric measurements, we analyzed the continuous variables using two approaches: direct comparison and stratification into categorical data based on predetermined cutoffs from previous studies in the Asian population. Except for total adiposity, we used optimal values determined by Youden’s index, as no prior cutoff was available. Univariable and multivariable Cox proportional hazards regression models will be constructed to quantify the association between OS and a priori determined set of covariates. Inter- and intra-reader reproducibility measurements for all CT measurements will be tested by interclass correlation coefficient (ICC).

In our dataset, only one parameter had missing values. Specifically, there were seven degraded CT images at the aortic arch level that prevented sarcopenia measurements from being performed, accounting for 4% of missing data. No other parameters had missing values. Therefore, this parameter was not included in multivariable Cox proportional hazard regression.

## Results

The baseline characteristics of the 172 enrolled patients are presented in Table 1. Almost all patients had adenocarcinoma (171 patients; 99.41%) and were in stage 4 (169 patients; 98.25%) with a mean age of 64.70 years (range from 34–91 years) and female predominance. The EGFR-TKI treatment included gefitinib (52.90%) and erlotinib (47.10%). The median OS time for the cohort was 17.57 months (95% CI 14.87–20.10 months).

**Table 1:** Baseline characteristics (Follow up at 18 months, N = 172).

Characteristics	Number of patients (N = 172)		p-value**
	Alive (N = 96)*	Death (N = 76)*	
Age at first diagnosis (years)	64.65 ± 10.57	64.76 ± 11.48	0.95
Sex			0.08
Male	27 (28.12%)	31 (40.91%)	
Female	69 (71.88%)	45 (59.21%)	
Body weight (Kg)	57.67 ± 11.83	54.19 ± 11.10	0.05
Height (cm)	157.45 ± 7.98	159.87 ± 8.20	0.05
BMI (Kg/m^2^)	23.25 ± 4.45	21.10 ± 3.44	0.007
Underweight (<18.5)	8 (8.33%)	19 (25.0%)	0.006
Normal (18.5–24.9)	62 (64.58%)	44 (57.89%)	
Obesity ( ≥25)	26 (27.08%)	13 (17.11%)	
ECOG			0.47
1	94 (97.92%)	73 (96.05%)	
2	2 (2.08%)	3 (3.95%)	
3	0	0	
4	0	0	
Smoking	34 (35.42%)	33 (43.42%)	0.34
Underlying disease***	59 (61.46%)	39 (51.32%)	0.18
Hypertension	47	29	
Diabetes	16	14	
Dyslipidemia	29	12	
Co-cancer	6	2	
COPD	1	4	
Metastatic site	94 (97.92%)	75 (98.68%)	0.70
1 organ	63 (65.63%)	52 (68.42%)	
>1 organs	33 (34.37%)	24 (31.58%)	
Staging****			0.70
Locally advanced (stage 3)	2 (2.08%)	1 (1.32%)	
Advanced (stage 4)	94 (97.92%)	75 (98.68%)	
Pathology*****			0.26
Adenocarcinoma	96 (100%)	75 (98.68%)	
Squamous cell carcinoma	0	1 (1.32%)	
Treatment			0.80
Erlotinib	46 (47.92%)	35 (46.05%)	
Gefitinib	50 (52.08%)	41 (53.95%)	

BMI; body mass index, kg; kilogram, cm; centimeter, m; meter, ECOG; Eastern Cooperative Oncology Group, COPD; Chronic Obstructive pulmonary disease.

* Mean±SD for continuous data and number (percent) for categorical data.

** T-test for continuous data and Fisher exact test for categorical data.

*** Presence of at least one underlying disease.

**** TNM staging, 8th edition, according to the American Joint Committee on Cancer (AJCC).

***** Pathology from histology of primary tumor or metastatic lymph node.

After 18 months of treatment initiation, 76 patients had died (44.19%). The group that died had a lower baseline BMI (21.10 ± 3.44) compared to the group that survived (23.25 ± 4.45) (p < 0.001). However, there was no statistically significant difference in body weight and height (p = 0.05). Additionally, there were no statistical differences between the two patient groups in terms of age, sex, metastasis, ECOG status, smoking, underlying disease, staging, pathology, and treatment (Table 1).

All CT scans were conducted within a median of 1.3 (IQR 0.93–2.0) months prior to the start targeted therapy. Table 2 presents the results of univariable, and multivariable analyses of non-cancerous CT factors correlated with 18-month mortality. For adiposity measurement at the L1 level, low total adiposity, low SAT and SATI were found to be significantly associated with the death group compared to the surviving group, with respective hazard ratios (HR) of 1.00 (95% CI: 0.99–1.00; p = 0.006), 0.99 (0.98–0.99; p = 0.005) and 0.98 (0.97–0.99; p = 0.003), respectively. When applying cutoffs, low total adiposity, low SAT and SATI, but higher VSR, showed statistically significant differences in the death group compared to the surviving group, with respective HRs of 2.65 (1.68–4.18; p < 0.001), 1.95 (1.23–3.11; p = 0.005), 1.74 (1.10–2.78; p = 0.019), and 1.67 (1.06–2.62; p = 0.026), respectively. VAT, VATI and abdominal girth at the L1 level showed no significant differences between the two groups.

**Table 2:** Univariable and multivariable analysis of non-cancerous CT factors correlated with 18-month mortality.

Variables	Number of patients (N = 172)		Univariable analysis**		Multivariable analysis***	
	Alive (96)*	Death (76)*	Hazard risk (95% CI)	p-value	Hazard risk (95% CI)	p-value
**Adiposity at L1 level**						
Total adiposity	146.90 (104.57–209.84)	118.31 (61.86 – 173.35)	1.00 (0.99–1.00)	0.006	1.01 (0.95–1.07)	0.741
≤85 cm^2^****	15 (15.62%)	33 (43.42%)	2.65 (1.68–4.18)	<0.001	3.91 (0.11–0.62)	0.002
SAT	86.73 (52.13–121.39)	64.48 (28.81–98.08)	0.99 (0.98–0.99)	0.005	1.00 (0.91–1.10)	0.958
≤45 cm^2^	19 (19.79%)	29 (38.16%)	1.95 (1.23–3.11)	0.005	1.61 (0.67–3.88)	0.285
VAT	54.84 (30.82–97.82)	44.72 (22.19–79.18)	0.99 (0.99–1.00)	0.072	–	–
≤75 cm^2^	86 (69.35%)	34 (70.83%)	1.19 (0.72–1.97)	0.499	–	–
SATI	34.80 (20.03–53.32)	24.48 (10.58–40.42)	0.98 (0.97–0.99)	0.003	0.96 (0.86–1.08)	0.520
≤30 cm^2^/m^2^female, ≤ 40 cm^2^/m^2^ male	43 (44.79%)	47 (61.84%)	1.74 (1.10–2.78)	0.019	1.67 (0.80–3.50)	0.175
VATI	22.50 (12.26–37.99)	16.24 (9.08–29.89)	0.99 (0.99–1.00)	0.07	–	–
≤35 cm^2^/m^2^ Female, ≤ 44 cm^2^/m^2^ Male	74 (77.08%)	64 (84.21%)	1.39 (0.74–2.57)	0.298	–	–
VSR	0.62 (0.42–1.18)	0.77 (0.41–1.46)	1.07 (0.99–1.13)	0.065	1.02 (0.95–1.10)	0.545
>0.93 Female, > 1.33 Male	35 (36.46%)	40 (52.63%)	1.67 (1.06–2.62)	0.026	1.86 (0.98–3.51)	0.057
Girth	85.13 ± 8.82	83.47 ± 8.85	0.98 (0.95–1.01)	0.220	–	–
≤80 cm Female, ≤ 90 cm Male	41 (42.71%)	38 (50.00%)	1.25 (0.80–1.96)	0.330	–	–
**Sarcopenia and myosteatosis at L1 level**						
MSA	74.58 ± 20.82	77.18 ± 23.83	1.00 (0.99–1.01)	0.310	–	–
<73.7 cm^2^ Female, < 116.2 cm^2^ Male	79 (82.29%)	58 (76.32)	1.27 (0.75–2.16)	0.371	–	–
SMD	46.42 ± 7.99	47.25 ± 6.69	1.00 (0.98–1.03)	0.546	–	–
<33.17 HU Female, < 37.42 HU Male	7 (7.29%)	4 (5.26%)	1.34 (0.49–3.68)	0.563	–	–
SMI	29.88 ± 7.09	29.81 ± 7.62	1.00 (0.97–1.03)	0.861	–	–
<27.5 cm^2^/m^2^ Female, < 39.2 cm^2^/m^2^Male	57 (59.38%)	47 (61.84%)	0.91 (0.57–1.44)	0.679	–	–
**OTHERS**						
Emphysema	8 (8.33%)	10 (13.16%)	1.39 (0.71–2.70)	0.331	–	–
Type of emphysema			0.59 (0.25–1.40)	0.231		
Paraseptal	3 (3.13%)	5 (6.58%)				
Centrilobular	3 (3.13%)	4 (5.26%)				
Mixed	3 (3.13%)	1 (1.32%)				
Grading of emphysema			1.40 (0.79–2.50)	0.252		
Mild	6 (6.25%)	5 (6.58%)				
Moderate	3 (3.13%)	3 (3.95%)				
Very severe	0	2 (2.63%)				
BMD	198.03 ± 57.82	200.05 ± 59.97	1.00 (0.99–1.00)	0.752	–	–
< 90 HU	1 (1.04%)	3 (4.00%)	0.47 (0.15–1.50)	0.206	–	–
MPA/AO	0.84 ± 0.13	0.86 ± 1.21	3.14 (0.35–26.13)	0.190	–	–
> 1	7 (7.29%)	7 (9.21%)	0.82 (0.37–1.77)	0.608	–	–
MPA size	25.38 ± 3.68	27.28 ± 4.11	1.09 (1.04–1.16)	0.001	1.14 (1.07–1.21)	<0.001
>29 mm	14 (14.58%)	24 (31.58)	2.23 (1.23–4.04)	0.005	3.10 (1.81–5.28)	<0.001

SAT; subcutaneous adipose tissue, VAT; visceral adipose tissue (VAT, cm^2^), SATI; subcutaneous adipose tissue index, VATI; visceral adipose tissue index, VSR; visceral-to-subcutaneous fat ratio, MSA; muscle surface area, SMI; skeleton muscle index, SMD; skeleton muscle density, BMD; bone mineral density, MPA; main pulmonary artery, AO; Aorta diameter, cm^2^; square centimeters, cm; centimeter, m^2^; square meters, HU; Hounsfield unit, mm; millimeters, CI; confidence interval.

* Mean ± SD for normal distribution data or median and IQR for non-normal distribution data.

** Cox regression analysis.

*** Cox regression analysis adjusted by BMI.

**** Only total adiposity was stratified by optimal values using Youden’s index of our data, but the remaining were used predetermined cutoffs according to the previous literatures.

For sarcopenic and myosteatotic measurement, there were no significant differences between the two groups at any level of measurement, including the L1 level (Tables 2 and 3). When applying the cut-off for diagnosing sarcopenia and myosteatosis at the L1 level, there was still no significant difference between the two groups (Table 2). An enlarged MPA size demonstrated HR of 1.09 (1.04–1.16; p = 0.001) and 2.23 (1.23–4.04; p = 0.005) when the size is > 29 mm. However, the MPA to aorta ratio did not show statistical significance (p = 0.190). Additionally, BMD and emphysema did not reach statistical significance (Table 2).

**Table 3:** Univariable analysis of sarcopenic and myosteatotic CT factors at T12, T10, T5, and the aortic arch levels correlated with 18-month mortality.

Variables	Number of patients		Univariable analysis	
	Alive (124)*	Death (48)*	Hazard risk (95% CI)	p-value**
Sarcopenia at T12 level				
MSA (cm^2^)	63.94 ± 20.21	67.29 ± 20.04	1.01 (0.99–1.02)	0.216
SMD (HU)	46.75 ± 7.02	46.93 ± 6.31	1.00 (0.96–1.03)	0.930
SMI (cm^2^/m^2^)	25.61 ± 7.07	26.04 ± 6.47	1.01 (0.98–1.04)	0.587
Sarcopenia at T10 level				
MSA (cm^2^)	55.92 ± 16.96	57.56 ± 20.00	1.00 (0.99–1.02)	0.408
SMD (HU)	42.29 ± 6.50	42.79 ± 6.59	1.01 (0.97–1.04)	0.677
SMI (cm^2^/m^2^)	22.39 ± 5.79	22.20 ± 6.50	1.00 (0.96–1.04)	0.955
Sarcopenia at T5 level				
MSA (cm^2^)	20.18 ± 8.90	22.31 ± 11.62	1.01 (0.99–1.03)	0.154
SMD (HU)	48.28 ± 8.43	48.67 ± 9.26	1.00 (0.98–1.03)	0.847
SMI (cm^2^/m^2^)	8.04 ± 3.17	8.52 ± 3.79	1.03 (0.97–1.10)	0.310
Sarcopenia at aortic arch level***				
MSA (cm^2^)	20.88 ± 8.56	22.54 ± 12.33	1.01 (0.99–1.03)	0.251
SMD (HU)	48.93 ± 8.23	48.70 ± 10.90	1.00 (0.97–1.02)	0.811
SMI (cm^2^/m^2^)	8.33 ± 3.08	8.66 ± 4.20	1.02 (0.96–1.09)	0.465

MSA; muscle surface area, SMI; skeleton muscle index, SMD; skeleton muscle density, cm^2^; square centimeters, m^2^; square meters, HU; Hounsfield unit, CI; confidence interval\.

* Mean ± SD.

** Cox regression analysis.

*** Seven degraded CT images at the aortic arch level prevented sarcopenia measurement from being performed.

In the multivariable analysis adjusted by BMI (Table 2), among all significant factors from univariable analysis, total adiposity ≤85 cm^2^ and enlarged MPA size retained statistical significance (p = 0.002 for total adiposity and p < 0.001 for both actual MPA size and size > 29 mm). Low total adiposity (≤85 cm^2^) and a large MPA size (>29 mm) alone showed hazard ratios (HR) of 3.91 (95% CI: 1.63–9.40) and 3.10 (95% CI: 1.81–5.28) for death at 18 months, respectively. Among BMD, adiposity, degree of emphysema, pulmonary artery size, and MPA to aorta ratio, the values of inter-observer reliability were moderate for the degree of emphysema (ICC = 0.69, CI = 0.15 – 0.89), and good to excellent for the remaining parameters (ICC = 0.77 - 0.95) [35].

## Discussion

Our study showed that baseline non-cancerous CT parameters can help predict OS at 18 months in advanced NSCLC patients during first-generation EGFR-TKI treatment as a first line systemic treatment. In our study population, where 44.19% of patients deceased at 18 months after starting targeted therapy, an enlarged MPA size, low total adiposity, and low subcutaneous fat (lower SAT, lower SATI, and higher VSR) were prognostic factors that predicted poor survival outcome. Additionally, BMI was also predictive of survival outcome. A large MPA size (MPA size > 29 mm) or low total adiposity (total adiposity ≤ 85 cm^2^) alone, after adjusting for BMI, could also be used as predictors of death at 18 months. Interestingly, all sarcopenic and myosteatotic measurements at any level, including the L1 level, were not statistically significant.

Our study focuses on advanced NSCLC with EGFR mutation treated with first-generation EGFR-TKIs, which is different from most of the studies in the meta-analysis [11,36] that focus on NSCLC treated with chemotherapy, radiotherapy, or surgery and range from early to advanced stages. Early identification of advanced NSCLC patients with a poor response to the first- generation of EGFR-TKIs could facilitate tailored treatment and potentially enable the use of high-generation targeted therapy or combination therapy with chemotherapy or anti-angiogenesis, which has demonstrated a longer survival rate compared to the first-generation [17,37,38]. Identifying patient status can also enhance patient management, such as improving nutritional status and reducing treatment-related side effects. These findings could help physician focus on important factors to assess patients’ fitness before treatment initiation. All parameters in our study followed previously established literature with available cutoff values specific to the Asian population at each level [9].

Pulmonary hypertension (PH) has been identified as a poor prognostic factor for NSCLC survival, regardless of when it is diagnosed. [39–41]. Our findings support that an MPA size greater than 29 mm independently predicts death at 18 months. While we did not use echocardiography or pulmonary catheterization to diagnose PH, previous studies show that MPA size correlates with PH with up to 82% accuracy [42]. PH in NSCLC may arise from shared risk factors, lung cancer itself, or cancer treatments. Emerging evidence links PH in NSCLC to vascular remodeling, inflammatory cell accumulation, and pulmonary tumor thrombotic microangiopathy, which can hinder cancer treatment and cause delays in care [41,43].

**Fig 4:**
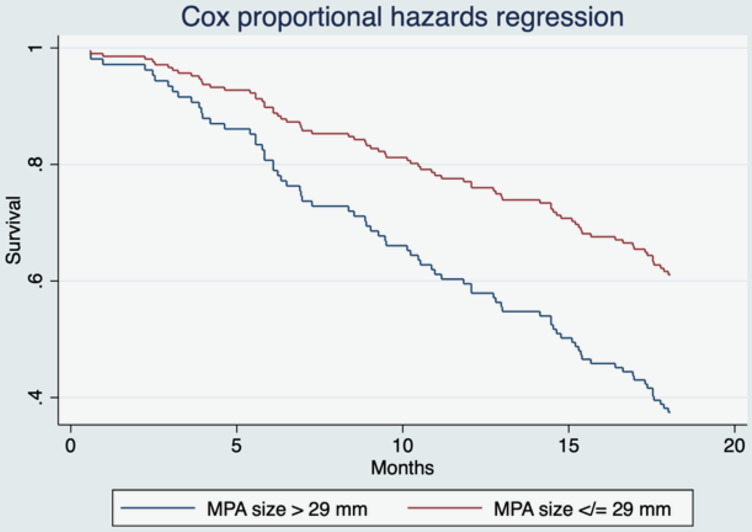
All-cause mortality of patients with MPA size > or ≤ 29 mm.

Interestingly, although an MPA-to-aorta ratio greater than 1 is indicative of PH, it was not significant in our study. This discrepancy may result from MPA size having higher sensitivity for PH compared to the MPA-to-aorta ratio, with respective values of 84% and 70% in previous study [44]. Another explanation is that with aging, the aorta typically enlarges [45], potentially resulting in a relatively larger aortic diameter compared to the MPA, leading to a lower MPA-to-aorta ratio and decreased sensitivity of this parameter. Given these factors, MPA size should be used as a prognostic indicator and adds value to routine chest CT scans as a tool for screening and monitoring PH in NSCLC patients [41] (Fig 4).

Adiposity measurements have shown conflicting results in lung cancer. A recent meta-analysis concluded that total, subcutaneous, and visceral adiposity were not linked to OS in lung cancer [26]. However, two studies found high subcutaneous adiposity and low visceral adiposity to be positive prognostic factors in NSCLC [28,46]. Our findings support this, showing that low SAT and low SATI, but not visceral fat, correlated with death at 18 months. These differences may be explained by the distinct metabolic effects of fat depots, which function as active endocrine organs secreting various adipokines that influence mortality risks differently [47]. Subcutaneous adipose tissue secretes more favorable adipokines, such as leptin and adiponectin, while visceral adipose tissue is linked to pro-inflammatory markers like IL-6 and TNF-α [47,48]. Increased visceral fat is strongly associated with a higher mortality risk, whereas subcutaneous fat is negatively associated with mortality [47]. Although total adiposity was significant in our analysis, we believed that subcutaneous fat likely played a major role in influencing this result. These findings suggest that adiposity measurements, particularly subcutaneous fat, could be reliable predictors in lung cancer, highlighting the importance of individualized nutritional support to optimize fat distribution and reduce inflammation. Further research on adiposity in lung cancer is warranted.

The level of measurement and cutoff value for adiposity need to be discussed. To our knowledge, there is no standard cutoff point for subcutaneous and visceral adiposity at the L1 level, but there is a correlation with measurements taken at the third lumbar vertebra (L3) level, as mostly studied [11]. Nitsche L et al. [49] demonstrated that both subcutaneous and visceral adiposity were 29.6% and 7.4% lesser at the L1 level compared to the L3 level, respectively. We used a cutoff of 45 cm^2^ for SAT and ≤30 cm^2^/m^2^ for females, and ≤40 cm^2^/m^2^ for males for SATI, as measured at the L3 level [28] and significant results remained at L1 level in our study. Our results support adiposity measurement at the L1 level with a cutoff like that at the L3 level as a potential predictor. Moreover, we demonstrated a cutoff of 85 cm^2^ for total adiposity at the L1 level with significant results. Further studies are warranted to confirm our findings in chest CT scans without including the L3 level.

Low BMI was identified as a poor prognostic factor in our findings. While studies have examined the relationship between BMI and survival outcomes, results are inconsistent. Some suggest low BMI as a poor prognostic factor in lung cancer [50,51], while others found that both low and high BMI worsen outcomes [52], and some report no significant differences [53]. BMI’s utility as a prognostic factor is limited, as it doesn’t account for fat distribution patterns or differentiate between body compositions, such as subcutaneous fat, visceral fat, and muscle [54]. More specific body composition measurements, such as imaging-based techniques, may better reflect a patient’s physical status than BMI, as supported by our study’s findings on the impact of different types of adiposities on OS [54,55].

Sarcopenia, characterized by a progressive loss of skeletal muscle volume and quality, can be diagnosed through CT measurements of muscle volume (MSA, SMI) and myosteatosis (SMD) [56]. In our study, sarcopenic and myosteatotic measurements at chest CT levels (excluding L3) showed no association with survival outcomes, contrasting with prior meta-analyses [36,57]. Lin TY et al. [36] found low SMI at the L3 level to be a poor prognostic factor in lung cancer, while Alexio GFP et al. [57] linked low SMD at L3 to poor survival across various cancers. Heterogeneity in study design, including differences in cutoff points, measurement levels, study populations, and ethnicities, may explain these conflicting results and pose challenges for cross-study comparisons [41]. Few studies have included advanced NSCLC patients, who are prone to cancer-induced cachexia, with sarcopenia rates ranging from 45.6% to 80% [58,59]. Our study found a similar prevalence (60%). Since the L3 level is not typically included in routine chest CT scans for lung cancer, our findings highlight limitations in using sarcopenia measurements for advanced lung cancer patients without L3-level data.

Our study has a few limitations. Firstly, the retrospective data collection from a single institution may introduce bias and result in missing data. However, we encountered only 7 instances of missing data for sarcopenia measurements at the aortic arch level. As a tertiary care center, our study population consists of advanced and complex cases, which may limit the generalizability of our results. Secondly, certain potential measurements, such as quantitative assessment of emphysema severity, intermuscular adipose tissue (IMAT) and intramuscular adipose tissue content (IMAC), which has shown significant correlation with survival outcomes in lung cancer were not conducted in this study [60]. However, this method is not practically feasible and requires additional software. Finally, despite using widely accepted cutoffs based on previous literature for each parameter, the absence of established cutoff points for sarcopenia and myosteatosis at some levels may affect the results. Ethnicity may influence sarcopenia and myosteatosis cutoff points; for instance, there is a lack of cutoff values for total adiposity measurements at the L1 level in studies involving Asian populations. However, we addressed this by analyzing continuous data alongside categorized data using cutoffs to mitigate this effect. Additionally, integrating these significant non-cancerous CT parameters with clinical and laboratory data, along with traditional cancer-related CT findings, could aid in developing a comprehensive prognostic scoring system. Further multicenter studies with larger populations are necessary to validate our findings and refine the scoring system for broader clinical application.

## Conclusion

Baseline chest CT parameters are valuable predictors of 18-month mortality in advanced NSCLC patients treated with first-generation EGFR-TKIs. In addition to tumor characteristics, non-cancerous factors such as enlarged MPA size, low total adiposity, and low subcutaneous fat (lower SAT, SATI, and higher VSR) emerge as key indicators of poor survival. MPA size (>29 mm) or low total adiposity (≤85 cm^2^) alone are strong predictors of mortality, emphasizing the need for early intervention. Routine chest CT scans should evaluate these markers to identify high-risk patients, facilitating more personalized care through closer monitoring, nutritional support, and consideration of higher-generation EGFR-TKIs or combination therapies. The lack of significant correlation between sarcopenia or myosteatosis (excluding L3) and survival, in these advanced NSCLC patients with a high prevalence of sarcopenia, highlights the importance of MPA size and adiposity as critical prognostic factors.

## Supporting information

S1 File List of patient characteristics and survival outcome. (XLSX)
